# Inverse ZrO_2_/Cu as a highly efficient methanol synthesis catalyst from CO_2_ hydrogenation

**DOI:** 10.1038/s41467-020-19634-8

**Published:** 2020-11-13

**Authors:** Congyi Wu, Lili Lin, Jinjia Liu, Jingpeng Zhang, Feng Zhang, Tong Zhou, Ning Rui, Siyu Yao, Yuchen Deng, Feng Yang, Wenqian Xu, Jun Luo, Yue Zhao, Binhang Yan, Xiao-Dong Wen, José A. Rodriguez, Ding Ma

**Affiliations:** 1 Beijing National Laboratory for Molecular Sciences, College of Chemistry and Molecular Engineering and College of Engineering and BIC-ESAT Peking University, Beijing, 100871 China; 2 Chemistry Department, Brookhaven National Laboratory, Upton, NY 11973 USA; 3 Institute of Industrial Catalysis, State Key Laboratory of Green Chemistry Synthesis Technology, College of Chemical Engineering, Zhejiang University of Technology, Hangzhou, 310032 China; 4 State Key Laboratory of Coal Conversion, Institute of Coal Chemistry, Chinese Academy of Sciences, Taiyuan, China; 5 National Energy Centre for Coal to Liquids, Synfuels China Co. Ltd, Beijing, China; 6 Beijing Advanced Innovation Center for Materials Genome Engineering, Industry-University Cooperation Base between Beijing Information S&T University and Synfuels China Co. Ltd, Beijing, China; 7 Department of Chemical Engineering, Tsinghua University, Beijing, 100084 China; 8 Materials Science and Chemical Engineering Department, State University of New York at Stony Brook, New York, NY 11794 United States; 9 Center for Electron Microscopy, Tianjin University of Technology, Tianjin, 300384 China; 10 X-ray Science Division, Advanced Photon Source, Argonne National Laboratory, Argonne, Lemont, IL 60439 USA

**Keywords:** Heterogeneous catalysis, Chemical engineering, Nanoparticles

## Abstract

Enhancing the intrinsic activity and space time yield of Cu based heterogeneous methanol synthesis catalysts through CO_2_ hydrogenation is one of the major topics in CO_2_ conversion into value-added liquid fuels and chemicals. Here we report inverse ZrO_2_/Cu catalysts with a tunable Zr/Cu ratio have been prepared via an oxalate co-precipitation method, showing excellent performance for CO_2_ hydrogenation to methanol. Under optimal condition, the catalyst composed by 10% of ZrO_2_ supported over 90% of Cu exhibits the highest mass-specific methanol formation rate of 524 g_MeOH_kg_cat_^−1^h^−1^ at 220 °C, 3.3 times higher than the activity of traditional Cu/ZrO_2_ catalysts (159 g_MeOH_kg_cat_^−1^h^−1^). In situ XRD-PDF, XAFS and AP-XPS structural studies reveal that the inverse ZrO_2_/Cu catalysts are composed of islands of partially reduced 1–2 nm amorphous ZrO_2_ supported over metallic Cu particles. The ZrO_2_ islands are highly active for the CO_2_ activation. Meanwhile, an intermediate of formate adsorbed on the Cu at 1350 cm^−1^ is discovered by the in situ DRIFTS. This formate intermediate exhibits fast hydrogenation conversion to methoxy. The activation of CO_2_ and hydrogenation of all the surface oxygenate intermediates are significantly accelerated over the inverse ZrO_2_/Cu configuration, accounting for the excellent methanol formation activity observed.

## Abstract (web summary)

Enhancing the intrinsic activity and space time yield of Cu based heterogeneous methanol synthesis catalysts is one of the major topics in CO_2_ hydrogenation. Here the authors develop a highly active inverse catalyst composed of fine ZrO_2_ islands dispersed on metallic Cu nanoparticles.

## Introduction

An increase of CO_2_ emissions caused by the extreme utilization of fossil fuels has led to serious concerns upon the crisis of global warming, the rising of ocean levels and sea acidification^1–3^. One of the most promising ways to mitigate the negative effects of excessive CO_2_ emissions is to convert the pollutant back into value-added chemicals and fuels, achieving a carbon neutral system by utilizing the CO_2_ as a carbon source^4–6^. The methanol economy^7^, advocated by George A. Olah^8^, proposes that the use of methanol or dimethyl ether can probably replace fossil fuels in the energy storage and ground transportation in the future^9–11^. Recent development of more efficient catalysts for the methanol to olefin/gasoline (MTO and MTG) reactions has also made the methanol a potential key compound for the manufacture of bulk chemicals^12,13^. Therefore, intensive research and effort have been devoted to finding high-performance catalysts for CO_2_ hydrogenation to methanol which may not have standard metal-oxide configurations or the typical copper and zinc oxide components of commercial catalysts^14–17^.

Copper-based catalysts have been widely investigated in the synthesis of methanol through CO_2_ hydrogenation^17–19^. In previous studies, it has been shown that Cu/ZrO_2_ systems exhibited promising catalytic performance^20^. Compared with other oxide supports, zirconia has been proven to be superior in promoting the catalytic performances of Cu-based catalysts in methanol synthesis through CO_2_ hydrogenation, especially due to its capability of inhibiting byproducts such as CO and methane^21^. Structural and mechanistic investigations suggested that the Cu-ZrO_2_ interface played a vital role in the selective formation of methanol, which facilitate the formation, further hydrogenation and desorption of the active surface intermediates^22–25^. To maximize the Cu-ZrO_2_ interface, it has been reported that amorphous ZrO_2_ was able to accommodate Cu in its lattice exhibiting much better performances than the crystalline tetragonal ZrO_2_ and monoclinic ZrO_2_ references^26,27^. Theoretical calculations even suggested that ZrO_2_ did not need to be presented as a bulk material in the catalyst^21^. In a study, isolated ZrO_x_ motifs dispersed on high surface area silica were able to promote the methanol synthesis activity and selectivity of supported Cu domains^28^.

These findings motivated us to investigate the CO_2_ hydrogenation over catalysts with a ZrO_2_/Cu inverse configuration in which the domains of zirconia only covered a small fraction of the metallic copper surface. At small coverages of zirconia, one can expect special structural and electronic properties not seen in the bulk material plus strong oxide↔copper interactions. Although being rarely investigated in powder catalysts, the inverse oxide/metal configuration has been shown to enhance interfacial reactivity in well-defined CeO_x_/Cu(111) and ZnO/Cu(111) model surfaces^29,30^ used as catalysts for the water-gas shift and methanol synthesis reactions. Furthermore, a ZnO/Cu configuration has been proposed as the actual active phase in a Cu/ZnO/Al_2_O_3_ powder catalyst^31^. In principle, an optimization of the oxide component in such an inverse oxide/metal configuration could lead to a better activity and selectivity for methanol synthesis^32,33^. Here, we move away from ZnO and work with a multivalent oxide component. In this article, combining catalytic tests and in-situ characterization, we show that ZrO_2_/Cu catalysts exhibit excellent performance in methanol synthesis, validating the idea of using inverse oxide/copper configurations aiming at a rational design of efficient catalysts for the CO_2_ to CH_3_OH conversion.

## Results

### The structural characterization of the ZrO_2_/Cu catalysts

**Fig. 1:**
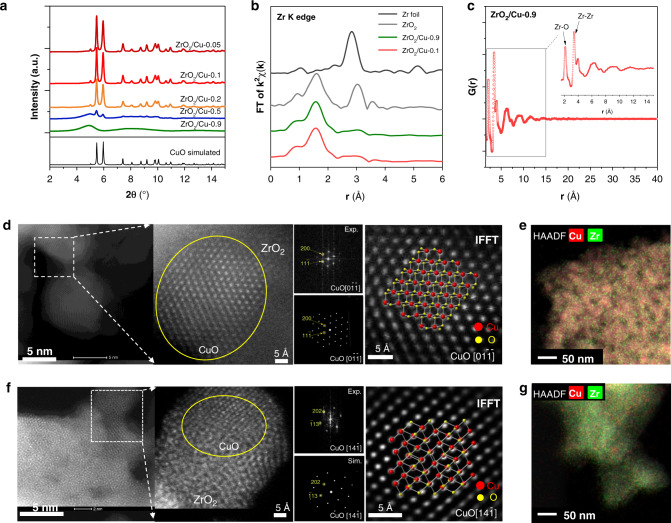
Structure characterization of the ZrO_2_/Cu-x catalysts. **a** XRD patterns of the calcined ZrO_2_/Cu-0.05 (dark red), ZrO_2_/Cu-0.1 (red), ZrO_2_/Cu-0.2 (orange), ZrO_2_/Cu-0.5 (blue) and ZrO_2_/Cu-0.9 (green) catalysts (wavelength of the incident X-ray, *λ* = 0.24125 Å). **b** EXAFS profiles of Zr K edge of ZrO_2_/Cu-0.1 (red) and ZrO_2_/Cu-0.9 (green) catalysts with Zr foil (black) and ZrO_2_ standards (gray) as references. **c** PDF structural analysis of the calcined ZrO_2_/Cu-0.9 catalyst. High-resolution HAADF-STEM images/FFT patterns with simulated results and the EDS elemental mapping of the ZrO_2_/Cu-0.1 **d**, **e** and ZrO_2_/Cu-0.9 **f**, **g** catalysts, respectively. The atomic structures of CuO [0$\bar 1\bar 1$] and [14$\bar 1$] are superimposed on the inversed FFT (IFFT) images. The scale bar in the **e** and **g** are 50 nm. In elementary mapping images, Zr and Cu are in green and red, respectively.

A series of catalysts with a ZrO_2_/Cu-x composition (where x is the weight percentage of ZrO_2_ in the sample and had values of 0.05, 0.1, 0.2, 0.5, and 0.9) were synthesized by using an oxalate co-precipitation (CP) method (for details of this approach see corresponding text in Methods and Supplementary Table 1)^34^. The powder diffraction profiles of the synthesized catalysts after calcination are shown in Fig. 1a. As no crystalline zirconia phases has been observed in any of the ZrO_2_/Cu-x catalysts, it was clear that the obtained ZrO_2_ species through the oxalate CP method were probably in an amorphous state. Zr K edge X-ray adsorption fine structure (XAFS) spectroscopy was collected to identify the local structure of the well dispersed ZrO_2_ nanoparticles. Since the Zr K edge FT-EXAFS spectra of both the ZrO_2_/Cu-0.1 and the ZrO_2_/Cu-0.9 catalysts showed similar R space structure with very small intensities of the second nearest neighbor Zr–Zr coordination, it is likely that the zirconia domains in the different ZrO_2_ loading catalysts have a similar geometrical structure (Fig. 1b). The pair distribution function (PDF) of the total x-ray diffraction of the amorphous ZrO_2_/Cu-0.9 catalyst was also collected to understand the structure of ZrO_2_ particles in the Zr rich catalyst (Fig. 1c). Based on the PDF profile, the nearest Zr-O pair distance is 2.13 Å and the nearest Zr–O–Zr pair appeared at 3.42 Å^35^, which correlates well with the Zr K edge EXAFS fitting results (Supplementary Fig. 1, Supplementary Table 2). The intensity of the oscillation of the PDF profile damped very quickly, and disappeared at ~12 Å, indicating that most of the ZrO_2_ particles had a size around 1.2 nm. When NaOH was used as the precipitating substance, the diffraction patterns of the ZrO_2_ could be observed for the resulted ZrO_2_/Cu-0.9-NaOH catalyst (Supplementary Fig. S2), indicating that the oxalate CP method tends to generate zirconia domains with a small size and amorphous structure, which is different from the traditional NaOH precipitation method. The structural information for the ZrO_2_/Cu-0.1 catalyst and ZrO_2_/Cu-0.9 catalyst was further confirmed by high-resolution scanning transmission electron microscopy (HR-STEM) and energy dispersive spectroscopy (EDS)-elemental mapping images (Fig. 1d–g). The local fast Fourier Transformation (FFT)^36^ derived from the high-angle annular dark field (HAADF)-STEM images suggested that the CuO in the ZrO_2_/Cu-0.1 catalyst consist of monoclinic crystals (ICSD 69094, space group: C 1 2/c 1) oriented along the [0$\bar 1\bar 1$] direction (Fig. 1d). However, the ZrO_2_ was present as amorphous species which decorated the surface of the CuO particles (around 15–20 nm), as no lattice fringes of ZrO_2_ could be resolved in the STEM images. The EDS elemental mapping images in Fig. 1e demonstrate that the ZrO_2_ component is highly dispersed on the CuO substrate. The decreasing percentage of Cu in the catalysts results in the transformation of Cu species from large particles (ZrO_2_/Cu-0.1, around 15–20 nm) to small particles (ZrO_2_/Cu-0.9, around 1–2 nm, Fig. 1f–g), which is in good agreement with the X-ray diffraction (XRD) patterns (Fig. 1a). Based on the characterizations, ZrO_2_/Cu-0.1 catalyst is named as inverse catalysts as the Cu becomes the major component as a support in the catalyst. The term “inverse catalyst” is an analogue of the previous studies in the surface science research^30^, which describes the catalyst composed by oxide islands that only covered a small part of the metal surfaces. When the ZrO_2_ is the dominant component, such as the ZrO_2_/Cu-0.9 system, the catalyst will be recognized as conventional Cu/ZrO_2_-0.1 catalysts.

### Catalytic performance of ZrO_2_/Cu-x in CO_2_ hydrogenation

**Fig. 2:**
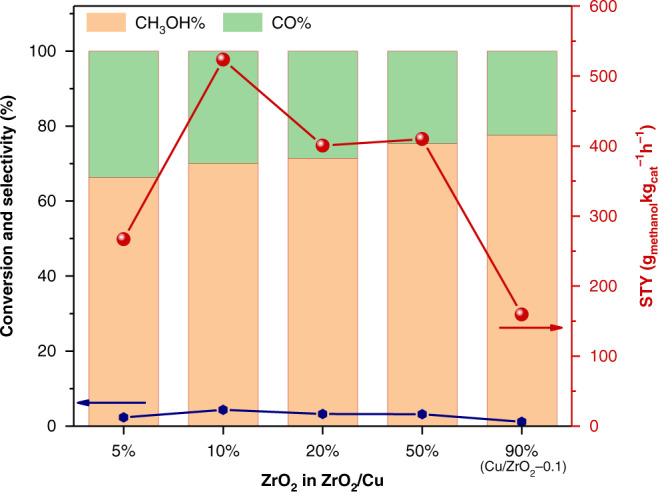
The catalytic performance of ZrO_2_/Cu catalysts. CO_2_ conversion (blue dotted line), product selectivity (methanol: orange bar, CO: green bar) and space time yield (STY) of methanol (red dotted line) as a function of the percentage of ZrO_2_ in the ZrO_2_/Cu catalysts. Reaction conditions for the catalytic test: WHSV = 48,000 mL g^−1^ h^−1^, T = 220 °C, CO_2_:H_2_ = 1:3, P = 3.0 MPa. All the performance data were collected at a CO_2_ conversion below 5%.

The catalytic activities of the ZrO_2_/Cu-x catalysts for methanol synthesis from CO_2_ hydrogenation were evaluated at 220 °C using a gas feed of CO_2_/H_2_ mixture with a 1/3 ratio at 3.0 MPa. Prior to the reaction, the pristine ZrO_2_/Cu-x catalysts were reduced by 5% H_2_ at 300 °C for 2 h. During the catalytic reaction, the CO_2_ conversion of the catalysts was kept below 5%, far away from the equilibrium concentration of methanol (19.4%, Supplementary Fig. 3) in order to approach the intrinsic activity. Among all the catalysts with different Zr/Cu ratio, the inverse ZrO_2_/Cu-0.1 catalyst showed the highest space time yield (STY) of methanol at a rate of 524 g_MeOH_kg_cat_^−1^h^−1^, 3.3 times higher than that of the ZrO_2_/Cu-0.9 (Cu/ZrO_2_−0.1) catalyst (159 g_MeOH_kg_cat_^−1^h^−1^) (Fig. 2). From this ZrO_2_/Cu-0.1 composition, the increasing or decreasing of the ZrO_2_ loading both triggered a reduction in the methanol formation rate, making the rate-to-ZrO_2_ loading relationship exhibit a volcano-like profile as shown in Fig. 2. What is more, the superior catalytic performance of ZrO_2_/Cu-0.1 is also comparable to that of a commercial Topsoe catalyst, which is well-known as one of the best methanol synthesis catalyst (Supplementary Fig. 4). Therefore, it can be concluded that the combination of the amorphous ZrO_2_ and Cu with a suitable Zr/Cu ratio is crucial for the construction of an excellent inverse catalyst for methanol synthesis from CO_2_. In the rest of the paper, the term of Cu/ZrO_2_-0.1 is used as the reference of tranditional catalyst.

### Structural understandings of the working catalysts

**Fig. 3:**
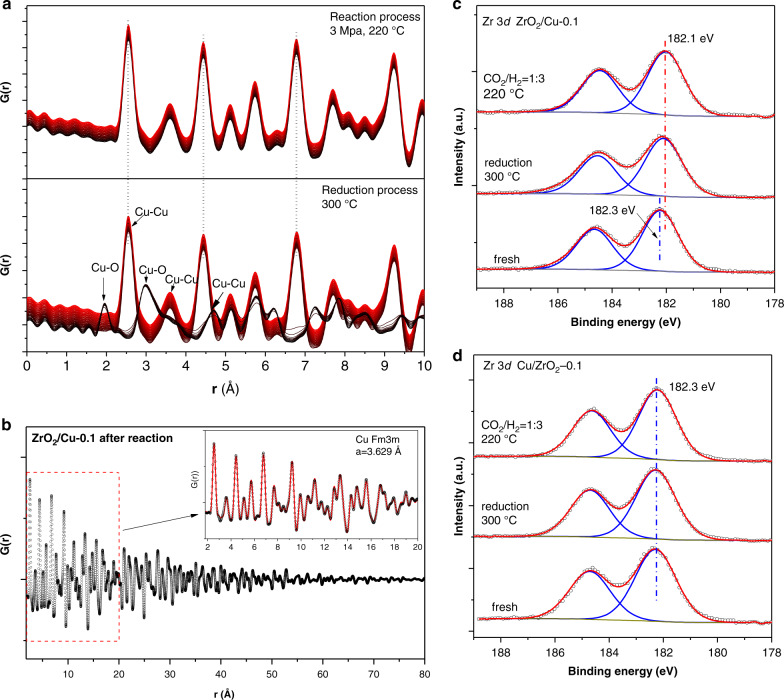
In situ characterizations for the ZrO_2_/Cu catalysts. **a** Pair distribution function in real space of ZrO_2_/Cu-0.1 catalyst in reduction condition (upper panel; reduced in the flow of 5% H_2_/He from room temperature to 300 °C, from black to red) and under reaction condition (bottom panel) (Reaction conditions: 2 ml min^−1^ CO_2_ + 6 ml min^−1^ H_2_, 3 MPa, 220 °C, reaction time: 60 mins; from black to red). **b** The PDF and fitting profiles (inserted figure) of the post reaction ZrO_2_/Cu-0.1 catalyst. The profile exhibited here was the last spectrum of obtained for the in situ reaction in part a. The AP-XPS Zr 3*d* region spectra of **c** ZrO_2_/Cu-0.1 and **d** Cu/ZrO_2_-0.1 catalyst.

To distinguish the structure characteristics of the inverse ZrO_2_/Cu-0.1catalyst and traditional Cu/ZrO_2_-0.1 catalyst which induced the different catalytic performances in CO_2_ hydrogenation, we performed systematic in situ characterizations on both the inverse and conventional catalysts (Fig. 3). An in situ XRD-PDF study was carried out to investigate the crystal structure of the ZrO_2_/Cu-0.1 catalyst under reduction and working reaction conditions of 3.0 MPa. Based on the profiles of synchrotron PDF (Fig. 3a), it could be confirmed that only the diffraction pattern of the CuO support was observable in the fresh sample. The reduction of the copper oxides into metallic copper took place at ~120 °C (Supplementary Fig. 5). During the 2 h reaction (220 °C), the intensity of crystalline Cu remained intact and no peak related with Cu_2_O was observed. According to the PDF fitting analysis, the Cu-Cu bond lengths are similar to those in bulk materials. Thus, no significant bond extension or contraction was observed during the reaction compared to the reduced sample (Fig. 3b), indicating that the structure of the Cu particles was stable under CO_2_ hydrogenation reaction conditions. In addition, the HR-TEM image and EELS element mapping of the spent ZrO_2_/Cu-0.1 catalyst (Supplementary Fig. 6) also demonstrate the ZrO_2_ remains as highly dispersed particles loaded on large Cu. No sintering or aggregation of zirconia has been resolved. As the surface composition of the catalyst under reaction environment is pivotal for chemical transformations, ambient pressure X-ray photoelectron spectroscopy (AP-XPS) was employed to determine the oxidation state of Zr in the inverse ZrO_2_/Cu-0.1 and traditional Cu/ZrO_2_-0.1 catalysts at elevated temperatures in the presence of H_2_ alone and CO_2_/H_2_ mixtures. As shown in Fig. 3c, the amorphous ZrO_2_ species loaded on Cu were partially reduced after 2 h exposure to H_2_ at 300 °C, with the binding energy of Zr 3*d* shifted to 182.1 eV, 0.2 eV lower than that of the fresh sample^37^. In contrast, no shift of the Zr 3*d* binding energy in the Cu/ZrO_2_-0.1 catalyst was observed, indicating that the reduction of ZrO_2_ species on the traditional catalyst was negligible (Fig. 3d). This phenomenon indicates that the highly dispersed ZrO_2_ domains over the Cu particles contain significant amount of O defects that can be formed in a reductive atmosphere (ZrO_2_/Cu-0.1). The oxygen vacancies probably serve as the active sites for CO_2_ capture and activation^17^.

### DRIFTS studies on the mechanism of CO_2_ hydrogenation

**Fig. 4:**
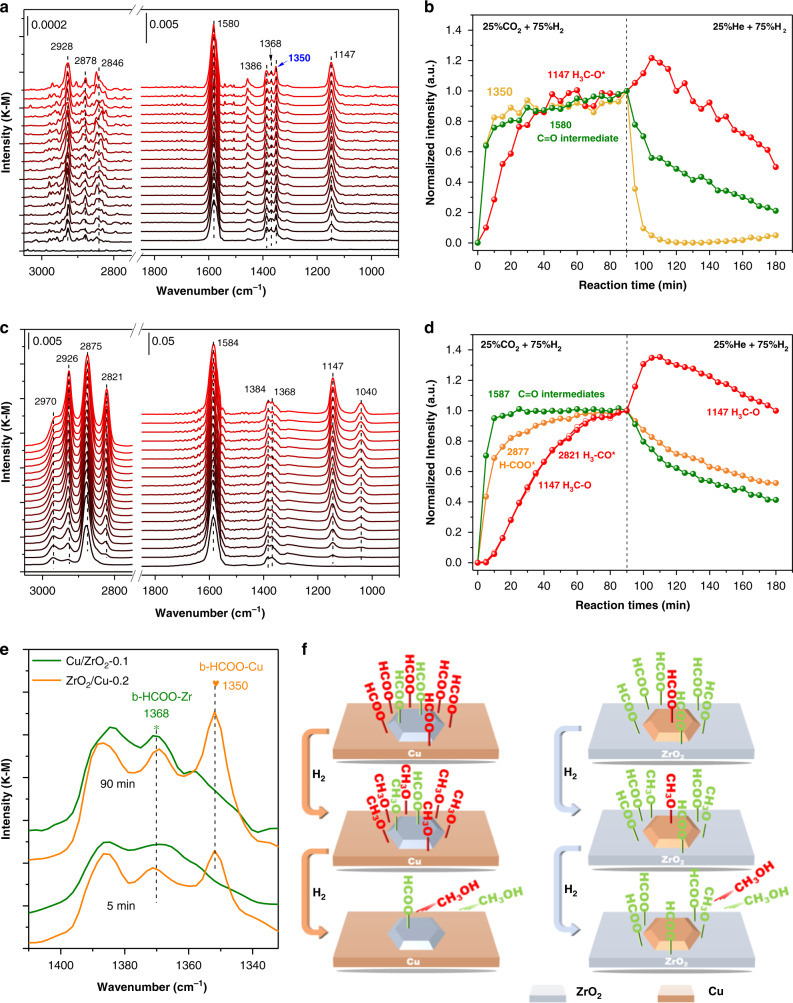
*Operando* DRIFTS observation. **a**, **c** In situ DRIFTS spectra of the CO_2_ + H_2_ reaction on ZrO_2_/Cu-0.2 and Cu/ZrO_2_-0.1 catalysts. The catalysts were exposed to 75% H_2_/25% CO_2_ (8 ml min^−1^) atmosphere at 220^o^C for 90 min (black to red lines). **b**, **d** Normalized intensities of the typical surface species including formate (2877 cm^−1^, orange dotted line), methoxy (2821 or 1147 cm^−1^, red dotted line), C=O (~1580 cm^−1^, green dotted line) and the peak of HCOO-Cu (1350 cm^−1^, yellow dotted line) species versus reaction time (first 90 mins in 75% H_2_/25% CO_2_ atmosphere at 220^o^C and the inlet was switched to 75% H_2_/25% He at 90 min and maintained at the same temperature for another 90 min) over ZrO_2_/Cu-0.2 and Cu/ZrO_2_-0.1 catalysts. The intensities of the species were normalized to the steady state of each corresponding IR band after 90 min reaction in 75% H_2_/25% CO_2_ atmosphere. **e** The enlarged spectra in the region of 1450~1320 cm^−1^ of ZrO_2_/Cu-0.2 (orange line) and Cu/ZrO_2_-0.1 (green line) catalysts. **f** The scheme illustrating the reaction behaviors of the HCOO-Cu and HCOO-Zr intermediates on inverse ZrO_2_/Cu and Cu/ZrO_2_ catalysts.

To elucidate the structural influences of the Cu-Zr composites on the surface adsorbates and intermediates was essential to the reaction mechanism, *operando* diffuse reflectance Fourier transform infrared spectroscopy (DRIFTS) studies were carried out under the typical CO_2_ hydrogenation atmosphere (Fig. 4, Supplementary Fig. 7). The ZrO_2_/Cu-0.2 was chosen as the model of the inverse catalyst instead of the ZrO_2_/Cu-0.1 catalyst, because the reflectance of ZrO_2_/Cu-0.1 catalyst is insufficient for performing IR measurements. As the reaction behavior of the ZrO_2_/Cu-0.2 is similar with that of ZrO_2_/Cu-0.1 catalyst, the IR studies will not be affected significantly. The vibration peak assignment for the major surface species is listed in Supplementary Table 3. The peaks located at 2970 and 2875, 1384 cm^−1^ are identified as the typical features of the adsorbed formate (H-COO*), while the bands centered at 1587 and 1368 cm^−1^ are attributed to the O-C-O vibration (denoted as C=O) of the formate species^16,21^. The IR bands of the methoxy groups (H_3_-CO*) are found at 2926, 2821 and 1147 cm^−1^^16^. Based on the DRIFTS spectra under steady state at 220 °C, the vibration peaks related with formate are generally very weak compared with the carbonates and methoxy species on the inverse ZrO_2_/Cu-0.2 catalyst (Fig. 4a), indicating the formate is a relatively active intermediate on the inverse catalyst. In contrast, it was obvious that there were intense signals of two major surface species, formate and methoxy, observed over the traditional Cu/ZrO_2_-0.1 catalyst (Fig. 4c). When tracking the intensity changes in the IR signals (2877, 2821 (1147) and ~1582 cm^−1^) which belong to the representative intermediates (formate, methoxy and C=O vibrations of formate and bidentate carbonates^38^) approaching the steady state after the catalysts were exposed to the reaction atmosphere (Fig. 4b, d, the left panel)^21,39^, the conversion of carbonates and formates into surface methoxy is much faster over the inverse ZrO_2_/Cu-0.2 catalyst. The intensity of the methoxy C-O vibration peaks at 1147 cm^−1^ approached the steady state within only 40 min after the exposure to the reactive atmosphere (Fig. 4b, left panel). In comparison, the carbonate, formate and the methoxy species reached steady state sequentially over the traditional Cu/ZrO_2_-0.1 (Fig. 4d). The methoxy intermediate reached the maximum by the end of 90 min, indicating that the hydrogenation of surface carbonates/formates into methoxy is a relatively slow step (Fig. 4d, left panel) over the conventional Cu/ZrO_2_-0.1 catalyst. The CO_2_ in the feed was cut off after the system reached steady state to observe the consumption of the surface species in pure H_2_^38^. The consumption of carbonates and formate species on the inverse ZrO_2_/Cu-0.2 catalyst is fast. Even under such a condition, the accumulation of methoxy is weak (less than 1.2 times of the equilibrium) and the remaining methoxy intermediate on the catalyst surface by the end of the 90 min test is only 40% of its original intensity (Fig. 4b, right panel), indicating the further reductive removal of methoxy is also a quick step on the inverse catalyst. On the contrary, on the traditional Cu/ZrO_2_-0.1 catalyst, with the intensity of carbonates and formate dropping (see Fig. 4d, right panel), the methoxy accumulated and its intensity reached 1.4 time higher than the equilibrium intensity before it was gradually consumed (the final intensity still above the initial value after 90 min reaction) (Supplementary Fig. 8). This phenomenon suggested that the hydrogenation of methoxy to methanol is kinetically even slower than the conversion of carbonates and formate to methoxy. Noticeably, except for the commonly appeared IR bands, a peak at 1350 cm^−1^ (HCOO-Cu)^40^ appeared on the inverse catalyst, which hardly appeared on the traditional catalyst (Fig. 4e, Supplementary Fig. 9). Based on the literature, the new band is attributed to the O-C-O vibration of the formatted species bonded on Cu or the interface, which is about 18 cm^−1^ lower than those adsorbed on the ZrO_2_ (bidentate formate^41^). The generation and consumption of HCOO-Cu were extremely fast under transient condition compared with the formate species on zirconia, which demonstrates a highly reactive reaction pathways existed on the inverse catalyst (Fig. 4b). The adsorption site of this intermediate, the metallic Cu, facilitates the hydrogenation conversion of the formate and enhances the turnover frequencies (TOF) of the oxygenates on the inverse catalysts. Summarizing the information of the in situ DRIFTS results, it could be deduced that the reason of the superior performance of the inverse configuration with optimized Zr/Cu ratio is that the oxygenate intermediates are able to be hydrogenated with a much higher rate than that on the traditional catalyst under similar reaction conditions (Fig. 4f).

**Fig. 5:**
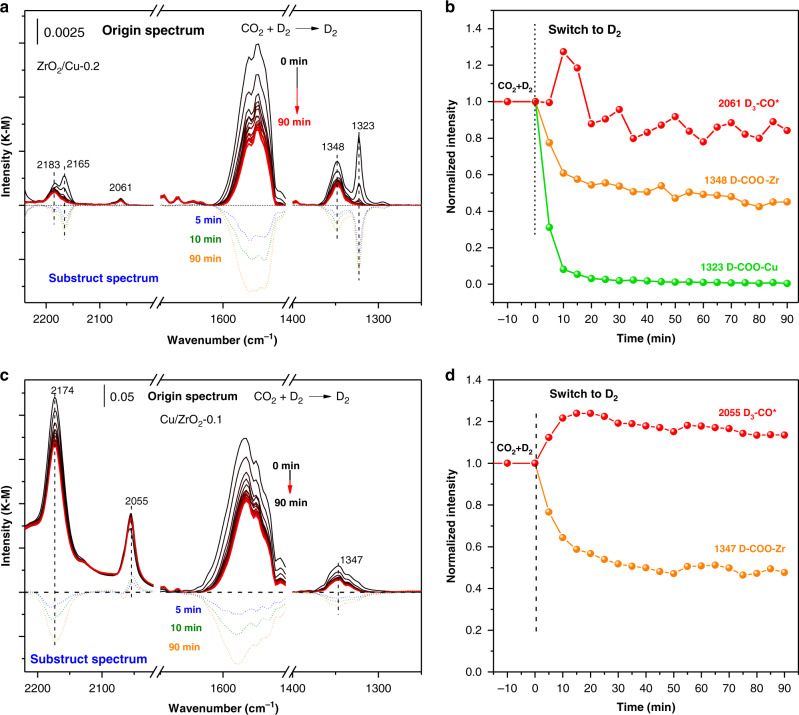
In situ DRIFTS observation of surface species with isotope exchange. **a**, **c** In situ DRIFT spectra of surface species of the ZrO_2_/CuO-0.2 and Cu/ZrO_2_-0.1 catalysts in the CO_2_ + D_2_ flow and subsequently cut off the CO_2_ in the D_2_ flow for 90 min (the upper panel, black to red lines). The spectra in the bottom showed the spectrum at 5, 10 and 90 min differentiate spectra to the one at 0 min, 5 min (blue dash line), 10 min (green dash line), 90 min (orange dash line). **b**, **d** The Normalized intensity of the IR peaks of the surface formate and methoxy as a function to reaction time on ZrO_2_/CuO-0.2 and Cu/ZrO_2_-0.1 catalysts. The infrared intensity of the species reaching steady state at 0 min of D_2_ or namely the 90 min in the CO_2_ + D_2_ flow has been arbitrarily scaled as 1 (Reaction conditions: 2 ml min^−1^ CO_2_ + D_2_ ml min^−1^ H_2_, 0.1 MPa, 493 K). D_3_-CO* (red dotted line), D-COO-Zr (orange dotted line; ʋ(C-D): 2174 cm^−1^, ʋ(C-O): 1348 cm^−1^), D-COO-Cu (green dotted line; ʋ(C-O): 1323 cm^−1^).

The H-D isotope effect was employed to further clarify the difference of IR bands on different configuration of ZrO_2_/CuO-0.2 and Cu/ZrO_2_-0.1 catalysts. The activated catalysts were firstly exposed to CO_2_ + D_2_ atmosphere for 90 min to achieve steady state (Supplementary Fig. 10), then the CO_2_ supply was cut-off (Fig. 5). As shown in the Fig. 5a and c, two new bands related with deuterium exchanged D-COO* and D_3_-CO* appeared at 2174 and 2056 cm^−1^. The symmetric O-C-O vibration of formate species was observed to shift from around 1386 to 1346 cm^−1^. The intermediate of HCOO-Cu was shifted from 1350 cm^−1^ to 1323 cm^−1^ of DCOO-Cu which is much clearer than in the spectra of CO_2_ + H_2_ reaction, as the previous one partially overlapped with another peak. The intensity of the DCOO-Cu over ZrO_2_/Cu-0.2 declined within 10 min after the cut-off of the CO_2_ feed (Fig. 5b). In comparison, the detectable surface species over Cu/ZrO_2_-0.1 declined gradually in the 90 min time scale after the cut-off of CO_2_ feed, while the intensity of the D_3_-CO* increases first before declining, due to the intermediate properties in the transformation from carbonates/formates to methanol (Fig. 5c, d). This comparative study of isotope effect further reveals that with the assistant of metallic Cu, the hydrogenation of the formate species (HCOO-Cu) accelerated, which probably explains the higher activity of the inverse catalyst.

### Theoretical calculations on CO_2_ adsorption

**Fig. 6:**
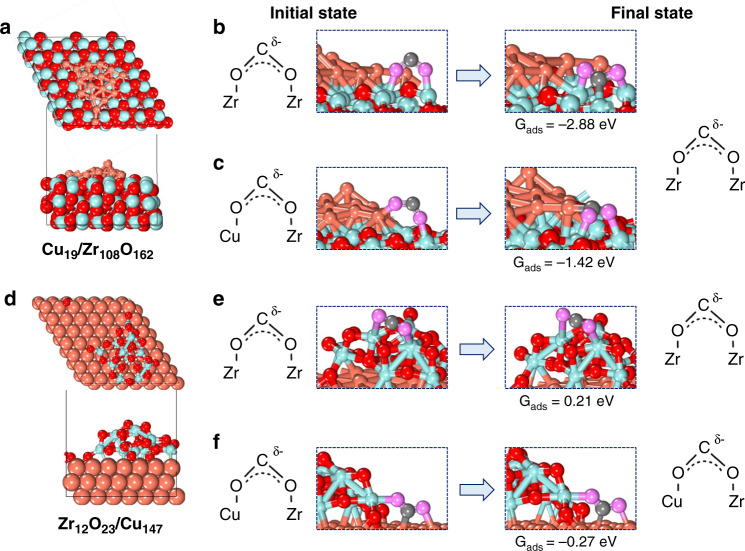
The configuration and adsorption free energy of CO_2_ on different models. The configurations of **a** Cu_19_/Zr_108_O_162_ presents Cu/ZrO_2_ catalyst, the initial states of CO_2_ adsorbed on Cu/ZrO_2_ **b** O-Zr, **c** O-Cu; the configurations of **d** Zr_12_O_23_/Cu_147_ presents ZrO_2_/Cu catalyst, the initial states of CO_2_ adsorbed on inverse ZrO_2_/Cu **e** O-Zr, **f** O-Cu. The salmon, cyan, black and red spheres represent Cu, Zr, C and O atoms, respectively. The pink spheres represent the O atoms in CO_2_ molecule.

The in-situ IR observation has revealed that the surface adsorbates on the inverse catalyst are partially different from the traditional configuration. Two models were used to simulated the conventional Cu/ZrO_2_ and inverse ZrO_2_/Cu catalysts (details seen Methods, and Supplementary Figs. 11 and 12). To obtain insight into this phenomenon, the adsorption of CO_2_ on conventional Cu/ZrO_2_ and inverse ZrO_2_/Cu catalysts was calculated to figure out the difference of formed configurations of CO_2_^*^ on these two surfaces (Fig. 6). The adsorption configurations of CO_2_^*^ are essential precursors for the formation of formate species. The calculation models of Cu/ZrO_2_ and inverse ZrO_2_/Cu are shown in Fig. 6a, d. The adsorption free energy was obtained at a temperature of 220 °C and a pressure of 3.0 MPa to simulate real reaction conditions. Based on the results of DRIFTS, two initial adsorption modes were established on Cu/ZrO_2_ and inverse ZrO_2_/Cu catalysts (Fig. 6b, c, e, f), respectively. On the Cu/ZrO_2_ model, the most favorable adsorption configuration of CO_2_ is both of the O atoms attached to the zirconia regardless of the initial state. Two of the most representative cases are shown in Fig. 6 and the adsorption free energy are −2.88 eV or −1.42 eV respectively (Fig. 6b, c). The bonding and configuration of this kind of CO_2_^*^ have also been reported in previously^41,42^. Therefore, the intermediate of COO^*^-Cu is not favored to be formed on the Cu/ZrO_2_ catalyst, going well with the results of DRIFTS (Fig. 4a). On the contrary, the two different CO_2_ adsorption initial states were evaluated on inverse ZrO_2_/Cu surface. The adsorption of bent CO_2_ at the interface (Fig. 6f) is more stable than that on ZrO_2_ particle (Fig. 6e) based on the calculated adsorption free energy (−0.27 eV vs 0.21 eV). The probability distribution of different adsorption configurations at 500 K were calculated by $P_m = \frac{1}{z}\exp \left[ {\frac{{ - \Delta E}}{{K_BT}}} \right]$ (Supplementary Table 4). These stable adsorption configurations on Cu/ZrO_2_ and inverse ZrO_2_/Cu can further convert into the formate intermediate in similar chemical bonding, indicating an adsorption configuration-driven mechanism.

In summary, we have shown that an inverse ZrO_2_/Cu catalyst composed of only 10% of zirconia loaded on metallic Cu particles showed a superior catalytic performance for the hydrogenation of CO_2_ to methanol when compared to a conventional zirconia supported Cu (Cu/ZrO_2_) catalyst. The best ZrO_2_/Cu catalyst displayed 3 times higher activity (524 g_MeOH_kg_cat_^−1^h^−1^) for the CO_2_ → CH_3_OH conversion. AP-XPS and DRIFTs characterizations showed that the ZrO_2_ species were in a highly reduced state and intermediates of formate and methoxy were formed and consumed on the inverse ZrO_2_/Cu much faster than on the Cu/ZrO_2_ configuration. The high activity is mainly ascribed to the formation of a highly reactive HCOO-Cu intermediate adsorbed on the metallic Cu component of the inverse ZrO_2_/Cu catalyst. The inverse configuration of ZrO_2_/Cu possesses distinct structure and catalytic properties and may be extended to other practical inverse catalysts. This study illustrates the power of using an inverse oxide/metal configuration and points to a new way for designing high-performance catalysts for CO_2_ hydrogenation to methanol.

## Methods

### Catalysts preparation

Synthesis of ZrO_2_/Cu-*x* (*x* = 0.05, 0.1, 0.2, 0.5, 0.9) catalysts. ZrO_2_/Cu-*x* catalysts were synthesized by co-precipitation method (CP). The oxalic acid was used as a precipitating agent. Taking the synthesis procedure of ZrO_2_/Cu-0.1 catalyst as an example: 0.02 mol Cu(NO_3_)_2_ and 0.002 mol Zr(NO_3_)_4_ precursors were dissolved in 100 mL ethanol. The mixed solution of precursors was then added into 0.5 M oxalic acid in ethanol solution under vigorous stirring. After 30 min reaction, the resultant solid was separated by centrifugation, followed by washing with ethanol and drying overnight. The obtained solid was calcined in the furnace at 400 °C for 2 h. The ZrO_2_/Cu-x catalysts with other x values were also prepared by a similar procedure. ZrO_2_/Cu-0.1-NaOH and ZrO_2_ /Cu-0.9-NaOH were synthesized by using NaOH as the precipitating agent.

The loading of the Zr and Cu was determined by ICP-OES.

### Catalytic evaluation

The performance evaluation for CO_2_ hydrogenation to methanol was performed in a high-pressure fixed-bed flow stainless steel reactor. 50 mg catalyst was diluted with quartz sand, and then packed into the stainless-steel tubular reactor. Prior to the catalytic measurements, the catalyst was reduced in a stream of 5% H_2_/N_2_ at 300 °C for 2 h under atmospheric pressure. Then, the temperature was cooled to 180 °C, and the reductive gas was replaced by the reaction gas of CO_2_ and H_2_ at a ratio of 1:3 (10 ml min^−1^ CO_2_: 30 ml min^−1^ H_2_). The reaction was performed with a pressure of 3.0 MPa. The catalytic activity test was taken at 180, 200 and 220 °C, and each temperature hold for 3 h. The reactants and products flowing out in the reactors were analyzed by an online gas chromatographer (GC-2014, Shimadzu) equipped with a TCD and a flame ionization detector (FID). The CO_2_ conversion and CH_3_OH selectivity were obtained from the GC data. All the conversion at different temperature are kept below than 5%. The conversion, selectivity and space time yield (STY) were defined in the following equation:

E1 $$Conv.({\mathrm{CO}}_2){\mathrm{\% }} = \frac{{n{\mathrm{CH}}_3{\mathrm{OH}} + n{\mathrm{CO}} + n{\mathrm{CH}}_4}}{{n{\mathrm{CO}}_2 + n{\mathrm{CH}}_3{\mathrm{OH}} + n{\mathrm{CO}} + {\it{n}}{\mathrm{CH}}_4}}$$

E2 $$Sel.({\mathrm{CH}}_3{\mathrm{OH}}){\mathrm{\% }} = \frac{{n{\mathrm{CH}}_3{\mathrm{OH}}}}{{n{\mathrm{CH}}_3{\mathrm{OH}} + n{\mathrm{CO}} + n{\mathrm{CH}}_4}}$$

E3 $$STY({\mathrm{CH}}_3{\mathrm{OH}})(g_{{\mathrm{CH}}_3{\mathrm{OH}}} \cdot kg_{{\mathrm{cat}}}^{ - 1} \cdot h^{ - 1}) = \frac{{F{\mathrm{CO}}_2 \ast Conv. \ast Sel.({\mathrm{CH}}_3{\mathrm{OH}}) \ast {\mathrm{32}} \ast {\mathrm{60}}}}{{{\mathrm{22}}{\mathrm{.4}} \ast m_{cat}}}$$

### Structural characterization

#### X-ray diffraction

The ex situ XRD diffraction patterns of tested catalysts were collected at the 17 BM beamline of the Advanced Photon Source (APS), Argonne National Laboratory (ANL) using a wavelength of the incident X-ray was 0.24121 Å. The fine powder of all the samples were loaded in the Kapton tube (1.0 mm OD). The powder diffraction images were collected using a flat panel amorphous silicon detector. The diffraction patterns were integrated using GSAS-II packages from corresponding images.

The in situ XRD characterization was performed at the same beamline with the ex situ XRD. The samples were placed into the high pressure in situ Clausen cell equipped with quartz tube (1.1 mm OD and 0.2 mm wall thickness). The reduction was performed at 300 °C in the flow of 5%H_2_/He. While, the reaction condition was carried out at 220 °C and in the presence of 3 MPa of 25% CO_2_/75% H_2_ mixture.

The PDF analysis of the in situ and ex situ XRD was performed using the GSAS-II packages. The composition of the samples is set based on the ICP-OES results.

#### X-ray adsorption fine structure

The Zr K edge and Cu K edge XAFS spectra of the catalysts were performed at BL14W beamline of the Shanghai Synchrotron Radiation Facility (SSRF) and also Beijing Synchrotron Radiation Facility (BSRF). Before the measurement, the samples were pressed into tablet.

All XAFS spectra were processed using the IFEFFIT package. The EXAFS oscillations were fitted according to back-scattering equation, using the FEFF models generated from crystal structures of ZrO_2_.

#### Ambient-pressure X-ray photoelectron spectroscopy

The AP-XPS measurement were carried out on a commercial SPECS AP-XPS chamber equipped with a PHOIBOS 150 EP MCD-9 analyzer (resolution: ~0.4 eV). The contaminated C1*s* (284.6 eV) was used for the energy calibration. The ZrO_2_/Cu-0.1, ZrO_2_/Cu-0.9 and amorphous CuO catalyst powders were pressed on an aluminum plate and then loaded into the AP-XPS chamber. The sample was reduced with 30 mTorr H_2_ at 300 °C for 2 h and cooled down to room temperature. Then 30 mTorr of H_2_ and 10 mTorr of CO_2_ were introduced into the reaction chamber. Zr 3*d*, Cu 2*p*, Cu LMM, O 1 *s*, and C 1 *s* XPS regions were collected at different temperatures (180 °C, 200 °C, 220 °C, 240 °C) under a reaction gas environment.

#### Diffuse reflectance infrared fourier transform spectroscopy

*Operando* DRIFTS measurements were performed by using an FTIR spectrometer (Bruker Vertex 80) equipped with a Harrick cell and an MCT detector, along with an RGA detector for the outlet gas analysis. The spectra were expressed in units of Kubelka-Monk (K-M). The ZrO_2_/Cu-0.2, 0.9 catalysts were reduced in 50 ml min^−1^ (H_2_ / He = 1:9) gas flow at 300 °C for 2 h and then cooled down to 220 °C. The gas flow was changed to 75% H_2_/25% CO_2_ (8 ml min^−1^, 0.1 MPa) at the same temperature and the spectra were collected simultaneously. After 90 min reaction in 75% H_2_/25% CO_2_ atmosphere, the inlet was switched to 75% H_2_/25% He (8 ml min^−1^) at the same temperature. At the same time, DRIFTS spectra were recorded to monitor the change of intensity of different surface species for another 90 mins. The signals were normalized basing on the intensity of signals after 90 mins reaction in 75% H_2_/25% CO_2_ (8 ml min^−1^) atmosphere.

#### TEM characterization

STEM experiments were carried out on a FEI Talos F200X electron microscope with a HAADF detector at 200 kV. EDX elemental maps were acquired from a Bruker super-X detection system on the Talos microscope.

The atomic coordination of the CuO was obtained from the Inorganic Crystal Structure Database (ICSD). Structural models and simulated electron diffraction patterns were generated by using CrystalMaker and SingleCrystal (CrystalMaker Software Ltd.).

#### Surface area measurement

N_2_ physisorption was performed on a Micromeritics ASAP-2020 instrument. The samples were first degassed in vacuum at 200 °C for 2 h before the measurement. The specific surface area and pore size distribution were calculated by using the Brunauer-Emmett-Teller (BET) method and Barrett-Joyner-Halenda (BJH) desorption branch, respectively.

The copper surface areas were determined by N_2_O titration on AutoChem II 2920 system equipped with a thermal conductivity detection (TCD) device. The samples were reduced in 5 vol% H_2_-Ar flow from room temperature to 500 °C and hold for 10 min. Then the catalysts were flushed with Ar flow and the temperature was decreased to 90 °C. Next, 2 vol% N_2_O in Ar was introduced to the reactor and hold for 30 min in a flow of 50 ml min^−1^. Finally, the catalysts were cooled to room temperature under Ar flow to conduct the second TPR run. The copper metallic surface area was calculated by assuming 1.4 × 10^19^ copper atoms/m^2^ and a molar stoichiometry N_2_O/Cus = 0.5, where the Cus means the copper atoms on the surface.

### DFT calculations

#### Computational models

For Cu/ZrO_2_ calculation model, a Cu_19_ cluster of truncated from Cu fcc structure was supported by a p(3 × 3) (−1 1 1) surface of monoclinic zirconia, this model contains 108 Zr, 162 O, and 19 Cu atoms as shown in Fig. 6(a), after the ab initio thermodynamics simulations of 10 ps, the Cu particle formed layered structure. During the optimization, 36 Zr and 54 O atoms were frozen while the others were relaxed. To model the inverse ZrO_2_/Cu surface, three Cu atomic layers of 7 × 7 (17.898 × 17.898 Å, Fig. 6b) were used. Based on ab initio atomistic thermodynamics, the formation of one oxygen vacancy is feasible in supported Zr_12_O_24_ particle (Supplementary Fig. S11). Therefore, the inverse model contains 147 Cu, 12 Zr, and 23 O atoms, where 49 Cu atoms was fixed, others atoms were relaxed. In all slab models, vacuum layers of 15 Å were applied to avoid the interactions between slabs in the z-direction.

It should be noticed that the model we used in theoretical calculation is just one of the possible and representative structures of the inverse and traditional catalysts, which is used to show the influence of the configuration on the adsorption of CO_2_ and support our in situ DRIFTS observation.

#### Computational methods

The DFT calculations were performed using the Vienna Ab Initio Simulation Package (VASP)^43^ with the frozen-core projector-augmented wave (PAW) method^44^. The generalized gradient approximation in the Perdew-Burke-Ernzerhof (GGA-PBE)^45^ function including D3 correction (Becke-Jonson damping)^46,47^ was employed for the exchange-correlation energy. An onsite Coulomb correction, *U*_eff_ = *U* − *J*, of 4.0 eV, was set for Zr 4*d* states^48,49^. A cutoff energy of 450 eV was selected for the plane-wave expansion. The convergence criteria for the force and electronic self-consistent iteration were set to 0.03 eV/Å and 10^−5^ eV, respectively. The Brillouin zone was sampled with a 1 × 1 × 1 for Cu/ZrO_2_ and ZrO_2_/Cu models (Supplementary Data 1 and 2). The adsorption free energy (G_ads_) were calculated based on G_ads_ = G_x/slab_ – [G_slab_ + G_x_], where G_x/slab_ is the total free energy of the slab with adsorbents after full relaxation, G_slab_ is the total free energy of the bare slab, and G_x_ is the total energy of the free adsorbents in the gas phase considering the temperature and gaseous pressure. Therefore, the more negative the G_ads_, the stronger the adsorption. In our calculations, the effect of temperature and pressure on free energy of solid is ignored, the corrected energy for adsorbents and free gas at *T* = 220 °C and *P* = 3 MPa was obtained by VASPKIT tool^50^. Ab initio molecule dynamics (AIMD) calculations were carried out at *T* = 500 K, the canonical (NVT) ensemble was used with a time step of 1 fs during 10 ps of a well-equilibrated trajectory. The number of oxygen vacancy in inverse catalyst model was calculated based on ab initio atomistic thermodynamics, the calculation details was same as stated in previous studies^51^.

## Supplementary information

Supplementary Information

Description of Additional Supplementary Files

Supplementary Data 1

Supplementary Data 2

## Data Availability

The data that support the plots within this paper and other finding of this study are available from the corresponding author upon reasonable request.
