# Supplementary material for: Inverse ZrO2/Cu as a highly efficient methanol synthesis catalyst from CO2 hydrogenation
Source: Nat Commun. 2020 Nov 13;11:5767. doi: 10.1038/s41467-020-19634-8 (PMC7666171; doi:10.1038/s41467-020-19634-8)
Supplement: Supplementary file 1 — Supplementary Information [file 41467_2020_19634_MOESM1_ESM.pdf]

Supplementary Information

Inverse ZrO_2/Cu as a highly efficient methanol synthesis catalyst from CO_2 hydrogenation

Congyi Wu[1†], Lili Lin[1,2,3†]*, Jinjia Liu[4,5†], Jinpeng Zhang[6], Feng Zhang[7], Tong Zhou[8], Ning Rui[2], Siyu Yao[2], Yuchen Deng[1], Feng Yang[1], Wenqian Xu[9], Jun Luo[8], Yue Zhao[1], Binhang Yan[6], Xiao-Dong Wen[4,5]*, José A. Rodriguez[2,7]*, Ding Ma[1]*

1. Beijing National Laboratory for Molecular Sciences, College of Chemistry and Molecular Engineering and College of Engineering and BIC-ESAT Peking University, Beijing 100871, China
2. Chemistry Department, Brookhaven National Laboratory, Upton, New York 11973, United States
3. Institute of Industrial Catalysis, State Key Laboratory Breeding Base of Green-Chemical Synthesis Technology, College of Chemical Engineering, Zhejiang University of Technology, Hangzhou 310032, China
4. State Key Laboratory of Coal Conversion, Institute of Coal Chemistry, Chinese Academy of Sciences, Taiyuan, China; National Energy Centre for Coal to Liquids, Synfuels China Co. Ltd, Beijing, China
5. Beijing Advanced Innovation Center for Materials Genome Engineering, Industry–University Cooperation Base between Beijing Information S&T University and Synfuels China Co. Ltd, Beijing, China.
6. Department of Chemical Engineering, Tsinghua University, Beijing 100084, China.
7. Materials Science and Chemical Engineering Department, State University of New York at Stony Brook, New York, 11794, United States
8. Center for Electron Microscopy, Tianjin University of Technology, Tianjin 300384, China
9. X-ray Science Division, Advanced Photon Source, Argonne National Laboratory, Argonne, Illinois 60439, United States

Supplementary Figures and Tables

Supplementary Table 1. The composition and surface area of different catalysts

**Catalysts**	**ZrO_2 loading [a] (wt %)**	**CuO loading [a] (wt %)**	**Total percentage**	**Surface area [b](m^2/g)**	**Cu metal area [c] (m^2/g)**
ZrO_2/Cu-0.05	6.9%	95.2%	102.1	28.8	68.2
ZrO_2/Cu-0.1	12.6%	90.3%	102.9	54.3	58.6
ZrO_2/Cu-0.2	22.9%	81.5%	104.4	72.6	34.6
ZrO_2/Cu-0.9	91.2%	8.4%	99.6	85.1	--

a. The concentration of Cu and Zr was measured by ICP-OES. wt (ZrO_2)% = C_{ICP}(Zr)*(123/91), wt (CuO)% = C_{ICP}(Cu)*(80/64). The total percentage was around 100%, which was in the reasonable deviation region.
b. BET method.
c. Calculated from N_2O titration method.

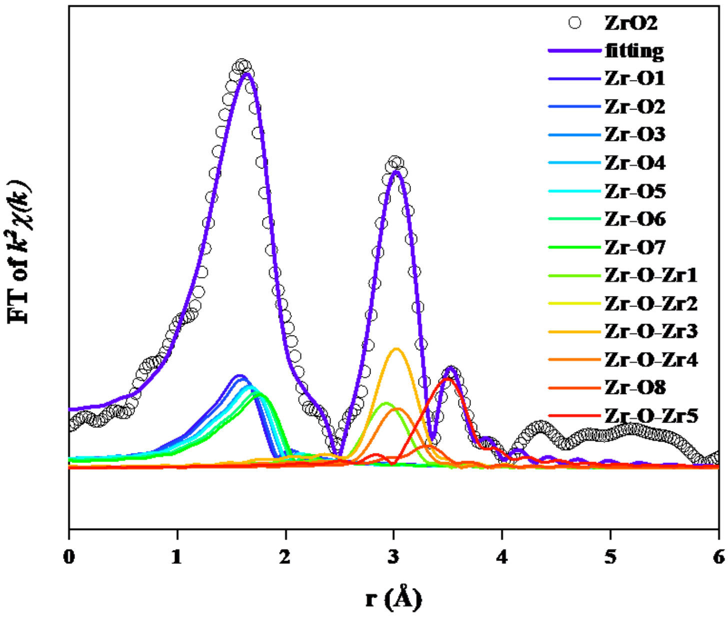

Supplementary Figure 1. The Zr K edge EXAFS plot of ZrO_2 reference.

Supplementary Table 2. The Zr K edge EXAFS fitting results of ZrO_2 reference.

Shell	C.N.	σ^2/*10^{-3}Å^2	E_0/eV	R/Å	R err/Å
Zr-O1	1			2.08	
Zr-O2	1			2.11	
Zr-O3	1			2.17	
Zr-O4	1	4.5	3.0	2.18	0.006
Zr-O5	1			2.19	
Zr-O6	1			2.25	
Zr-O7	1			2.28	
Zr-O-Zr1	1			3.37	
Zr-O-Zr2	2	8.1	-10.3	3.46	0.007
Zr-O-Zr3	2			3.46	
Zr-O-Zr4	1			3.47	
Zr-O8	1	10.0	9.9	3.76	0.022
Zr-O-Zr5	1			3.77	

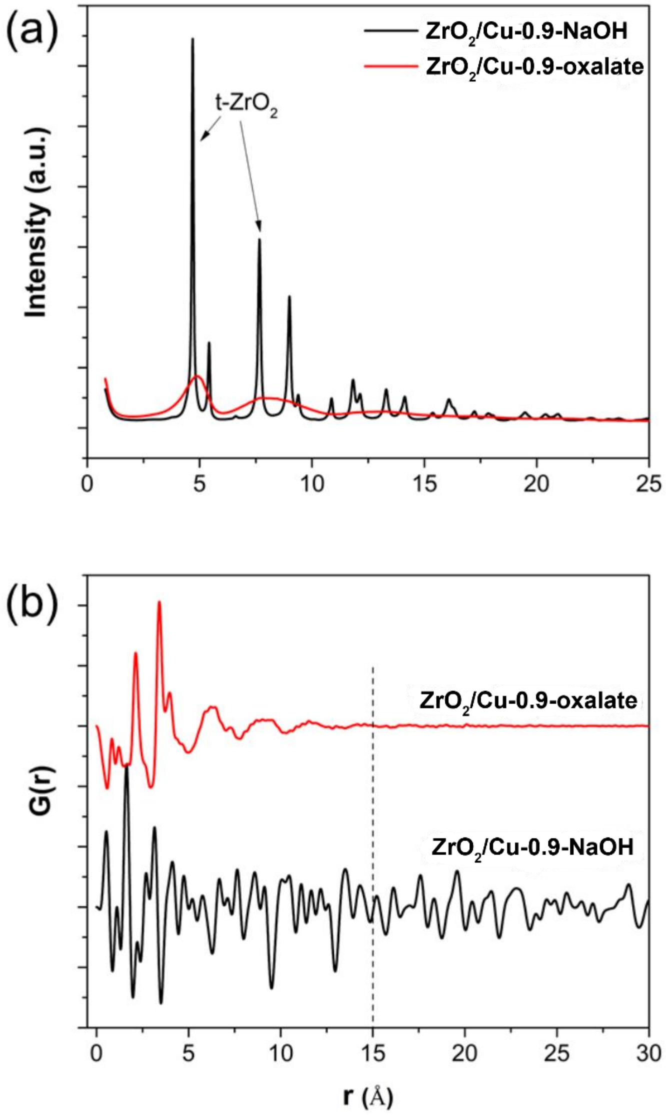

Supplementary Figure 2. The XRD profiles (a) and PDF fitting results (b) of ZrO_2/Cu-0.9 catalysts prepared with NaOH or oxalate as precipitating agent. The diffraction patterns of ZrO_2 can be observed when NaOH was used as the precipitation reagent. This result demonstrates the advantages of the oxalic acid precipitation method in preparing the fine dispersion of ZrO_2 domains.

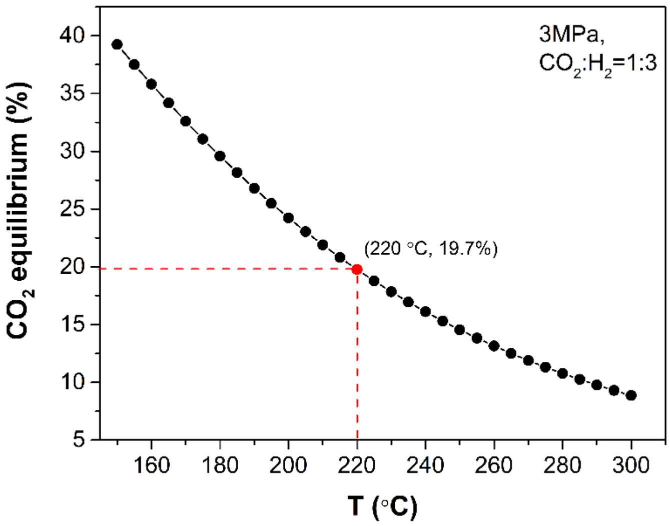

Supplementary Figure 3. The thermodynamic calculation on the CO_2 conversion equilibrium for CO_2 hydrogenation at pressure of 3.0 MPa with the initial state of $P(CO_2)/P(H_2)$ =0.75 MPa: 2.25 MPa. The CO_2 equilibrium conversion at 220°C is 19.7%.

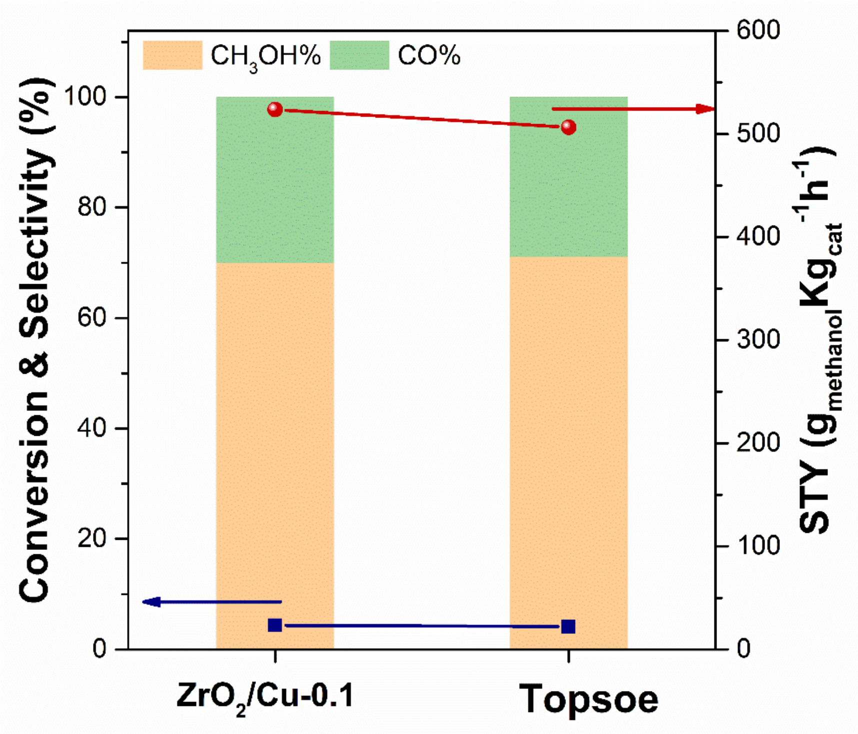

Supplementary Figure 4. The catalytic performance comparison of ZrO_2/Cu-0.1-oxalate and a Topsoe catalyst. Reaction conditions for the catalytic test: WHSV = 48,000 ml g^{-1} h^{-1}, T = 220 °C, CO_2:H_2 = 1:3, P = 3.0 MPa. All the performance data were collected at a CO_2 conversion below 5%.

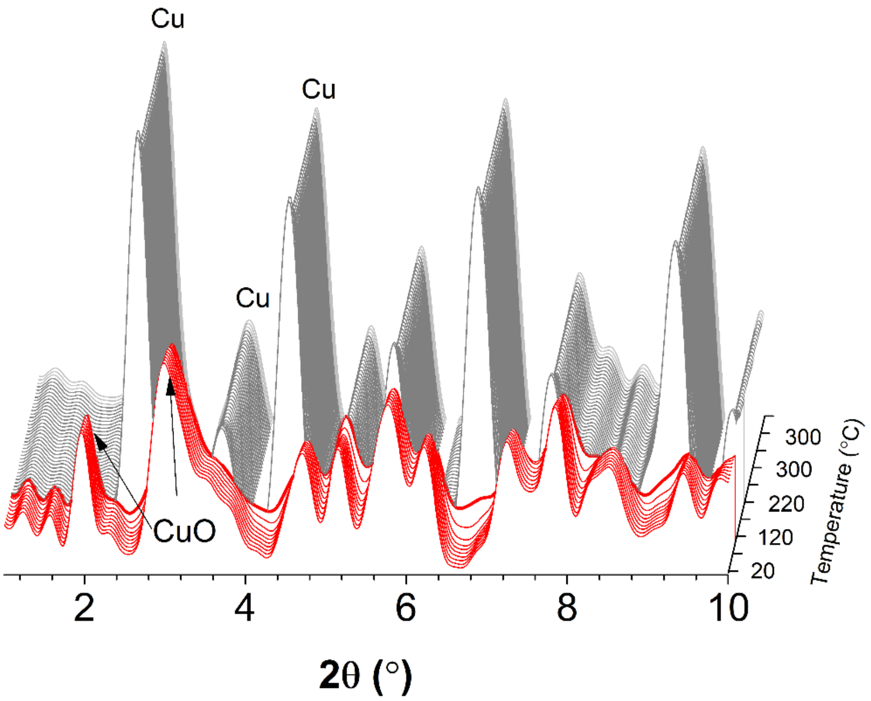

Supplementary Figure 5. The *in-situ* XRD characterization of the reduction process of ZrO_2/Cu-0.1 catalyst. The red lines indicated the Cu in ZrO_2/Cu-0.1 catalyst was in CuO state before reduction. The temperature of the phase transformation from CuO to Cu appeared at around 120 °C.

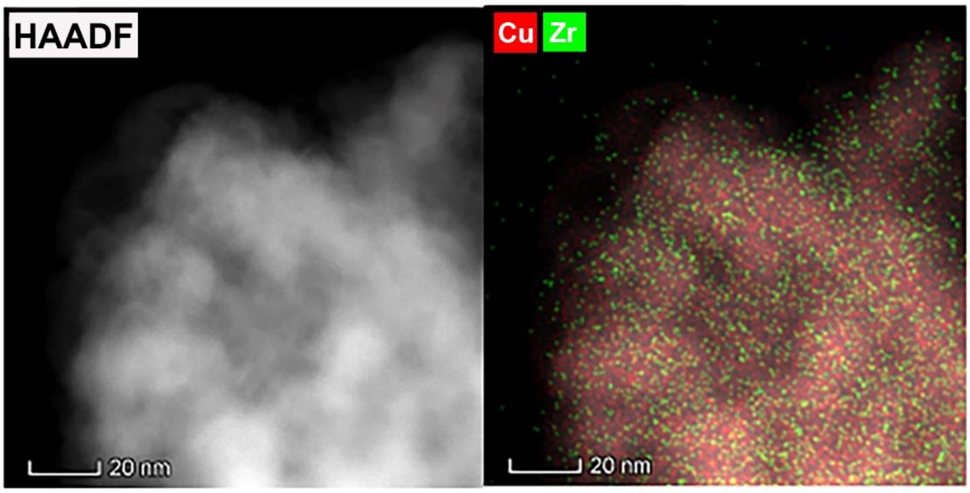

Supplementary Figure 6. The post ZrO_2/Cu-0.1 catalyst was characterized by HR-TEM. No notable aggregation of ZrO_2 particles was observed, indicating the good stability of the inverse catalyst.

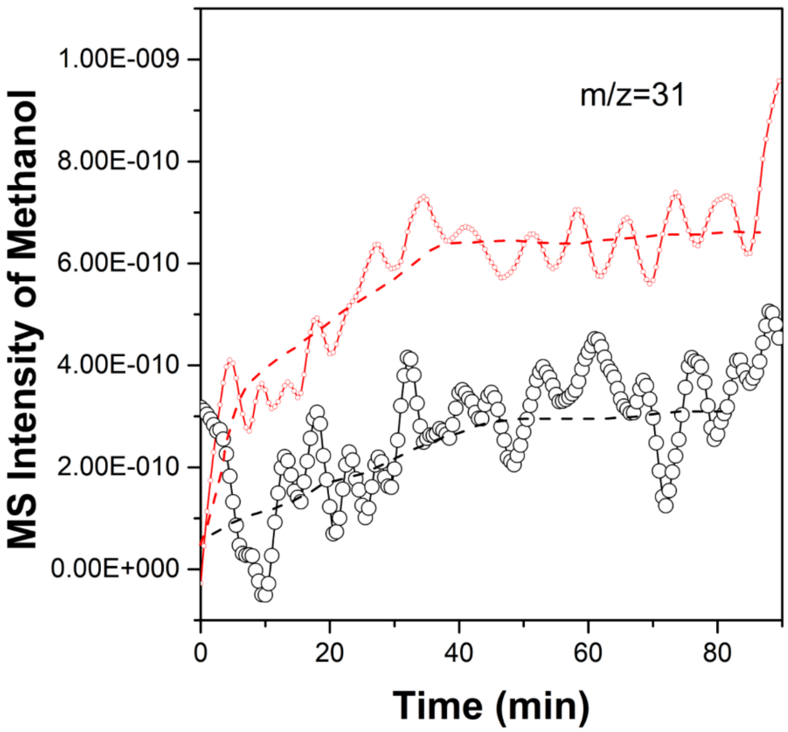

Supplementary Figure 7. The mass spectra of m/z=31 (methanol) of ZrO_2/Cu-0.1(red line) and Cu/ZrO_2-0.1 (gray line) in the *operando* DRIFTS characterization. The data was collected at the same time with Figure 4.

Supplementary Table 3. The assignment of the FTIR wavelengths of the surface species [1-7]

Surface species	Wavenumber (cm^{-1})	Assignment
methoxy	2926	$\nu_{as}(CH_3)$
	2821	$\nu_s(CH_3)$
	1147	ν(O-C) of b-OCH_3
	1040	ν(O-C) of t-OCH_3
Bidentate formate	2970	δ(CH)+ ν_{as}(OCO)
	2875	ν(CH)
	1584	ν_{as}(O-C-O)
	1543	ν_{as}(O-C-O) (HCOO-Cu)[7]
	1384	δ(CH)
	1368	ν_s(O-C-O)
	1350	δ(CH) (HCOO-Cu)[7]

Footnote: the biggest the difference between formate adsorbed on the Cu (HCOO-Cu) or Zr (HCOO-Zr) is the peak located at 1350 cm^{-1} based on the reference 7.

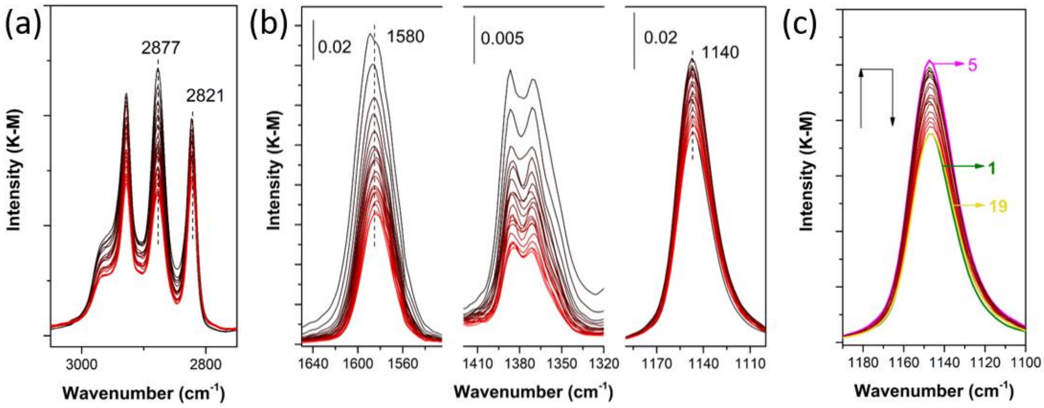

Supplementary Figure 8. ***In-situ* DRIFTS spectra of the He + H_2 reaction on Cu/ZrO_2-0.1 catalyst approaching steady state.** (a) the spectra in the range of 3200~2700 cm^{-1}, (b) the spectra in the range of 1650~1510 cm^{-1}, (c) the enlarged spectra change tendency of H_3CO* at 1140 cm^{-1}. The green, magenta, yellow lines mean the catalyst saturated with intermediates exposed to 75% H_2/25% He (8 mL/min) for 0, 15 and 90 min, representatively. The Cu/ZrO_2-0.1 catalyst was first exposed to CO_2 + H_2 for 90 min to reach the steady state (as shown in Figure 3f). The spectrum at 90 min was taken as the first spectrum as exposed to H_2/He for 0 min. Then, the catalyst was kept in 75% H_2/25% He (8 mL/min) atmosphere at 220°C for 90 min (black to red lines)

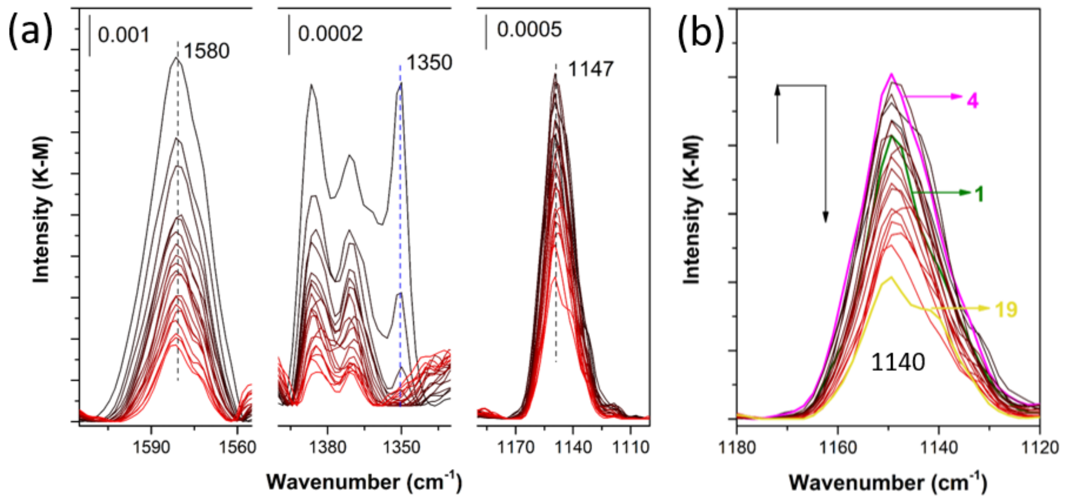

Supplementary Figure 9. *In-situ* DRIFTS spectra of the He + H_2 reaction on the ZrO_2/Cu-0.2 catalyst approaching the steady state. (a) spectra in the range of 1650~1100 cm^{-1}, (b) the enlarged spectra change tendency of H_3CO^* at 1140 cm^{-1}, the green, magenta, yellow lines illustrate the catalyst saturated with intermediates after being exposed to 75% H_2/25% He (8 mL/min) for 0, 15 and 90 min, representatively. The ZrO_2/Cu-0.2 catalyst was first exposed to CO_2 + H_2 for 90 min to reach the steady state (as shown in Figure 3d). The spectrum at 90 min was taken as the first spectrum as exposed to H_2/He for 0 min. Then, the catalyst was kept in 75% H_2/25% He (8 mL/min) atmosphere at 220°C for 90 min (black to red lines)

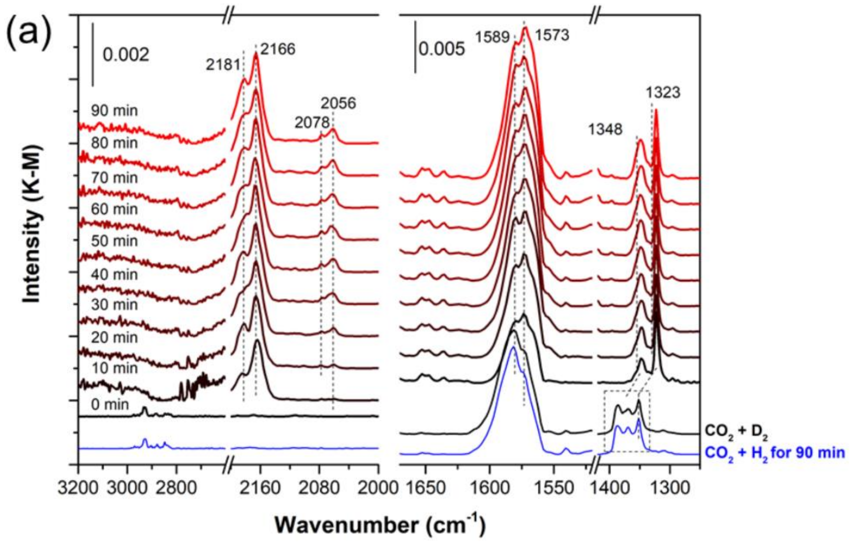

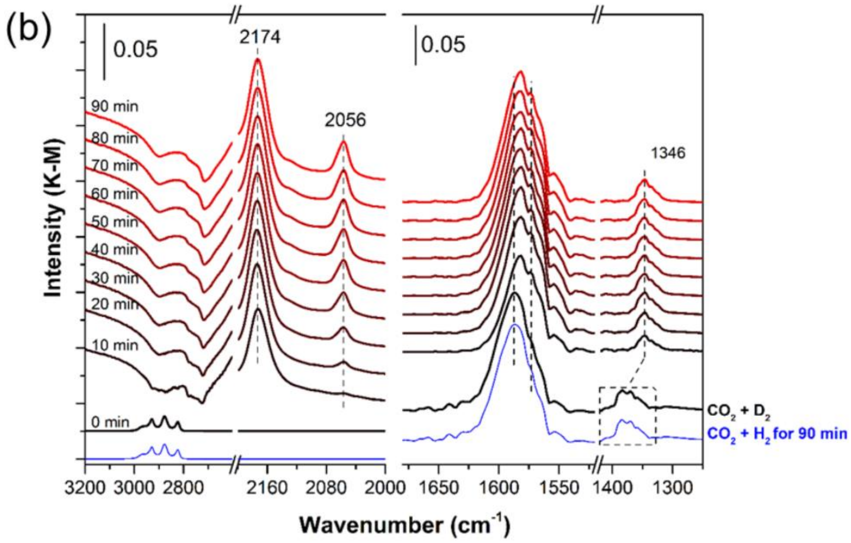

Supplementary Figure 10. *In-situ* DRIFTS characterization of surface species with isotope exchange over (a) ZrO_2/Cu-0.2, (b) Cu/ZrO_2-0.1 catalysts. The blue spectrum at the bottom of each figure is exposed to CO_2 and H_2 atmosphere for 90 min to achieve steady state, then cutting off H_2 and changing to D_2 for 90 min. During this process, the shift of the intermediates from H-C vibration to the D-C one is obvious, meanwhile, the intermediates with D-C vibration exhibited stronger intensity.

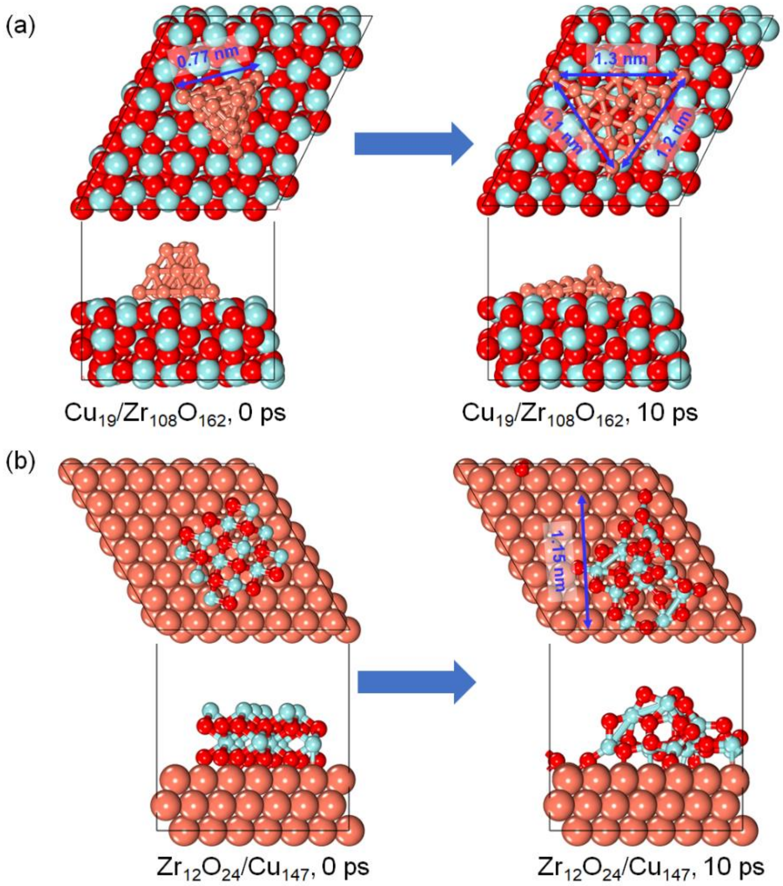

Supplementary Figure 11. Top and side views of initial (0 ps) and final (10 ps) structures during AIMD simulation of (a) $Cu_{19}/Zr_{108}O_{162}$, (b) $Zr_{12}O_{24}/Cu_{147}$ under 500 K.

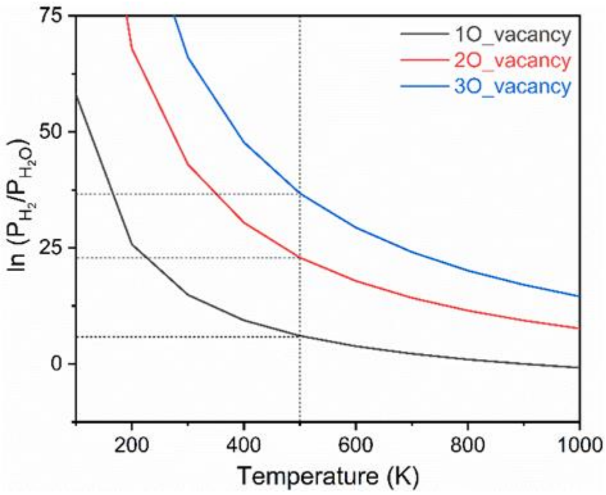

Supplementary Figure 12. The equilibrium phase diagram between different number of oxygen vacancies in $Zr_{12}O_{24}/Cu_{147}$ model. The lines represent the free energy of different number of oxygen vacancies formation (ΔG_r) is zero. The region under the lines represent the ΔG_r is positive. Oppositely, the region above the lines represent the ΔG_r is negative.

Supplementary Table 4 Probability distribution of different adsorption configurations at 500 K calculated by $P_m = \frac{1}{z} exp\left[\frac{-\Delta E}{K_B T}\right]$. The adsorption configurations (a)-(d) are corresponding as noted in Figure 6.

	Adsorption configurations	P_m
Cu/ZrO_2	(a)	1.0
	(b)	0
ZrO_2/Cu	(c)	0.18
	(d)	0.82

Supplementary References

[1] Bianchi, D., Gass, J.-L., Khalfallah, M. & Teichner, S. J. Intermediate species on zirconia supported methanol aerogel catalysts: I. State of the catalyst surface before and after the adsorption of hydrogen. *Appl. Catal. A: Gen.* **101**, 297-315 (1993).
[2] Bianchi, D., Chafik, T., Khalfallah, M. & Teichner, S. J. Intermediate species on zirconia supported methanol aerogel catalysts: II. Adsorption of carbon monoxide on pure zirconia and on zirconia containing zinc oxide. *Appl. Catal. A: Gen.* **105**, 223-249 (1993).
[3] Bianchi, D., Chafik, T., Khalfallah, M. & Teichner, S. J. Intermediate species on zirconia supported methanol aerogel catalysts: IV. Adsorption of carbon dioxide. *Appl. Catal. A: Gen.* **112**, 219-235 (1994).
[4] Bianchi, D., Chafik, T., Khalfallah, M. & Teichner, S. J. Intermediate species on zirconia supported methanol aerogel catalysts V. Adsorption of methanol. *Appl. Catal. A: Gen.* **123**, 89-110 (1995).
[5] Wang, J. et al. A highly selective and stable ZnO-ZrO_2 solid solution catalyst for CO_2 hydrogenation to methanol. *Sci. Adv.* **3**, e1701290 (2017).
[6] Kattel, S., Yan, B., Yang, Y., Chen, J. G. & Liu, P. Optimizing binding energies of key intermediates for CO_2 hydrogenation to methanol over oxide-supported copper. *J. Am. Chem. Soc.* **138**, 12440-12450 (2016).
[7] Fisher, I. A. & Bell, A. T. In-situinfrared study of methanol synthesis from H_2/CO_2 over Cu/SiO_2 and $Cu/ZrO_2/SiO_2$. *J. Catal.* **172**, 222-237 (1997).
