# Supplementary material for: Inverse ZrO2/Cu as a highly efficient methanol synthesis catalyst from CO2 hydrogenation
Source: Nat Commun. 2020 Nov 13;11:5767. doi: 10.1038/s41467-020-19634-8 (PMC7666171; doi:10.1038/s41467-020-19634-8)
Supplement: Supplementary file 2 — Description of Additional Supplementary Files [file 41467_2020_19634_MOESM2_ESM.pdf]

Description of Additional Supplementary Files

File Name: Supplementary Data 1
Description: Crystallology information file of Cu@ZrO2

File Name: Supplementary Data 2
Description: Crystallology information file of ZrO2@Cu
